# Gut microbiota influences *Plasmodium falciparum* malaria susceptibility

**DOI:** 10.1016/j.nmni.2025.101586

**Published:** 2025-04-14

**Authors:** Aly Kodio, Drissa Coulibaly, Safiatou Doumbo, Salimata Konaté, Abdoulaye Kassoum Koné, Souleymane Dama, Amadou Niangaly, Mamadou Lamine Tall, Ahmed Mohamed Konaté, Coralie L'Ollivier, A. Levasseur, Fadi Bittar, Abdoulaye Djimdé, Ogobara K. Doumbo, Didier Raoult, Mahamadou Ali Thera, Stéphane Ranque

**Affiliations:** a Aix Marseille Université, Service de Santé des Armées, RITMES, 19-21 Boulevard Jean Moulin, 13005, Marseille, France; b IHU Méditerranée Infection, 19-21 Bd Jean Moulin, 13005, Marseille, France; c Malaria Research and Training Centre-International Center for Excellence in Research (MRTC-ICER), Department of Epidemiology of Parasitic Diseases, Faculty of Medicine and Dentistry, Université des Sciences, des Techniques et des Technologies de Bamako, Point G, BP 1805, Bamako, Mali; d Aix Marseille Université, Assistance Publique-Hôpitaux de Marseille, MEPHI: Microbes, Evolution, Phylogénie et Infection, 19-21 Boulevard Jean Moulin, 13005, Marseille, France

**Keywords:** Gut microbiota, *Plasmodium falciparum*, Parasitemia, malaria attack, 16S metagenomics, ITS metagenomics, Malaria, Children, Mali

## Abstract

**Background:**

The gut microbiota has recently been associated with malaria susceptibility/resistance in animal models and humans. This study aimed to assess its influence on malaria attack and *Plasmodium* parasitemia in children living in a malaria-endemic area of Mali.

**Methods:**

Healthy children were enrolled in a 16-month cohort study in Bandiagara. Their gut bacteria and fungi community structures were characterized via 16S and ITS metabarcoding at enrolment. Clinicians monitored malaria attacks. Asymptomatic *Plasmodium* carriage was assessed by real-time polymerase chain reaction.

**Results:**

Of the 300 children, 107 (36 %) had at least one malaria attack, and 82 (27 %) had at least one episode of asymptomatic *Plasmodium* parasitemia. The gut bacterial community structure, but not the fungal community, was associated with susceptibility/resistance to both malaria attacks and asymptomatic *P. falciparum* parasitemia. Higher gut bacteria richness was independently associated with susceptibility to both asymptomatic parasitemia episodes and malaria attacks. 17 bacteria, and 7 fungi were associated with susceptibility to malaria attacks, and 8 bacteria, and 3 fungi were associated with resistance. 15 bacteria and 13 fungi were associated with susceptibility to asymptomatic *Plasmodium* parasitemia episodes, and 19 bacteria and 3 fungi were associated with resistance.

**Conclusion:**

Further studies are needed to confirm these findings, which point the way to strategies aimed at reducing the risk of malaria by modulating gut microbiota components in at-risk populations.

## 1 Introduction

Malaria is caused by a protozoan parasite, *Plasmodium* spp., which are transmitted to humans through the infesting bite of a female *Anopheles* mosquito. The highest malarial burden is clustered in Africa and the area south of the Sahara makes up 98 % of the 228 million cases worldwide each year. *Plasmodium falciparum* malaria is the most life-threating parasitic disease worldwide, despite the implementation of multiple control strategies. It causes 405,000 deaths or more each year and children under the age of five are the most vulnerable population, making up 67 % (272,000) of these deaths [1].

Malaria parasite carriage can progress as an asymptomatic infection, as uncomplicated malaria with symptoms as fever, headache, and chills, or as severe malaria including high parasitemia, severe anaemia, respiratory distress, and cerebral malaria (altered consciousness and seizures) [2,3]. However, factors have been described to be associated with malarial resistance, including ethnic and genetic factors, such as sickle cell traits (AS, C), Duffy negative blood group, Human Leukocyte Antigen group, and polymorphisms in immune response genes [[3], [4], [5], [6], [7], [8]]. In areas of intense malaria transmission, young children are more susceptible to malaria due to the loss of maternal antibody protection from 9 months. They present a high malaria morbidity and mortality. Older children, as a result of repeated malaria attacks, acquire a non-sterilising premunition that reduces the risk of severe malaria and associated case fatality [9,10]. The immune response to *Plasmodium* infection is partially known and the occurrence of malaria is variable from one individual to another and in the same individual [11,12]. This poses the difficulty of developing an effective malaria vaccine which is also linked to the complexity of the biology of *Plasmodium* and the arsenal that it has developed over the years to adapt to the immunity of its hosts [13,14].

Recently, the gut bacterial community has been pointed out as a protective factor against *Plasmodium* infection in humans. In a recent study, anti-α-gal antibodies were associated with protection against *Plasmodium* infection in humans and in the mouse model. Anti-α-gal antibodies, induced by the pathobiont *Escherichia coli* O86:B7 in the mouse gut, are cytotoxic to antigens on the surface of *Plasmodium* sporozoites, thus protecting mice from the transmission of *Plasmodium* infection by mosquitoes. The same study reported an association between anti-α-gal IgM levels and protection against malaria infection [15]. Also, natural anti-α-Gal IgG3 and IgG4 antibody levels were raised in children who had experienced no malaria attack within one year of follow-up, indicating evidence of protection against malaria infection in Mozambican children [16]. Beyond the transmission of *Plasmodium* infection, the gut microbiota has been associated with severe malaria infection. Lactobacillus and Bifidobacterium species in the gut have shown to play a protective role against *Plasmodium* infection by reducing the parasite load and attenuating the severity of malaria in mice [2]. Severe malaria infections alter the functional capacity of the microbiota, improving bacterial motility and amino acid metabolism in mice with a high parasite load compared to a mild infection [17]. Severe malaria infection can be modulated by the gut microbiota in genetically diverse mice and pregnant animals. The abundance of *Akkermansia muciniphila*, *Allobaculum*, *Lactobacillus* and S24-7 has also shown to be negatively correlated with parasite load [18]. Also, severe malaria infection in pregnant mice, which is a function of the composition of the intestinal microbiota, significantly influences foetal and postnatal outcomes [18]. Notably, the bacterial of the microbiota was associated with the risk of asymptomatic *Plasmodium* parasitemia occurrence and not of malaria attack [19]. However, no studies have yet established that the bacterial microbiota contributes towards protecting against malaria in humans, and the influence of the fungal microbiota on malaria has not yet been investigated. This study aimed to assess whether both bacteria and fungi communities in the gut were associated to the susceptibility/resistance against asymptomatic *Plasmodium* infection and malaria attacks in children living in a malaria-endemic area, Bandiagara (Mali).

## 2 Materials and methods

### 2.1 Ethical declaration

This study was approved by the Ethics Committee of the Faculty of Medicine of Mali (N°2017/133/CE/FMPOS). Each child included, and/or at least one of their parents or guardians gave their informed, written consent to participate in the study.

### 2.2 Patient recruitment and specimen collection

A longitudinal cohort study has been conducted from October 2017 to December 2018 in Bandiagara Malaria Project (BMP) clinical research centre in Mali. This period consisted of a season of low transmission from January to June and a season of seasonal and intense malaria transmission from July to December. The study involved children aged from six months to years who were not taking drugs with known antimalarial activity or antibiotics and who had no clinical symptoms of malaria. Recruited children have been monthly monitored for asymptomatic *Plasmodium* parasitemia and, whenever a child presented with malaria symptoms, for a possible malaria attack. A clinical examination and a thick blood film and a blood drop on blotting paper were collected at each of each time point visit.

Children faeces were collected at Day-0 in identified sterile jars and immediately placed at 4 °C. Hard stools were diluted v/v with 10X PBS (Phosphate-Buffered Saline pH 7.4, RNase-free) solution. Stool aliquots were distributed into 1 ml tubes, kept at 20 °C in Mali, then packed in dry ice and shipped to Marseille for further 16S and ITS metabarcoding and realt-time polymerase chain reaction (qPCR) detection of eukaryotic enteric pathogens.

### 2.3 Malaria case definitions

Malaria attack was defined by the detection of *Plasmodium* sp., at any density in at least one thick blood smear, and the presence of compatible clinical symptoms, including fever (body T°>37.5 °C) or other malaria-associated symptoms. The thick smear was stained with 5 % Giemsa and the number of parasites for 300 leukocytes was counted under a light microscope. Parasite density was estimated by calculating the number of asexual forms of *Plasmodium*/μl of blood under the assumption that the leukocyte count was 7500/μl. All children in whom a malaria attack was diagnosed were given antimalarial treatment according to the guidelines of the National Malaria Control Programme in Mali.

Asymptomatic *Plasmodium* parasitemia was defined by the detection *Plasmodium* sp. DNA by qPCR on blood samples collected on blotting paper during the monthly monitoring of asymptomatic children. The identified blotting papers were dried, sealed in sachets with desiccants and stored at room temperature. Total DNA was extracted using the EZ1 DNA tissue kit (Qiagen GmbH, Hilden, Germany). Blotting paper fragments were inserted into a 1.5 ml tube containing 350 μl of G2 lysis buffer and allowed to diffuse for 20–30 min. The tubes containing the blotting paper were incubated at 100 °C for 10 min and shaken briefly and then centrifuged at 10,000 g for 10 min, before removing 200 μl of the supernatant. A mixture of the supernatant with 10 μl of Proteinase K was incubated at 55 °C, for either 2 h or overnight. Total DNA was obtained using the EZ1 Advanced XL (QIAGEN Instruments Hombrechtikon, Switzerland) with the DNA card bacteria V 1.066069118 QIAGEN and the EZ1 DNA tissue kit according to the manufacturer's procedures. The extracted total DNA was stored at 4 °C and immediately used for the detection of *Plasmodium* species. The extracted DNA was analysed by qPCR using the CFX96TM and CFX384TM Real-Time PCR Detection Systems (BIO-RAD, Life Science, Marnes-la-Coquette, France) using the following probes and primers (Table 1). The amplification reaction consisted of 10 μL Master Mix (Roche Diagnostics GmbH, Mannheim, Germany), 0.5 μL of each primer, 0.5 μL of probe, 3 μL of distilled water, 0.5 μL of UDG and 5 μL of DNA for a total volume of 20 μL. The amplification programme followed consisted of 2 min at 50 °C and 5 min at 95 °C, followed by 40 cycles of 5 s at 95 °C and 1 min at 60 °C. The amplification solution without a DNA template was the negative control; the positive control was DNA from samples from patients in whom a *Plasmodium* sp. (*P. falciparum*, *P. ovale*, P. malaria or P. vivax) has been documented. Samples with a cycle threshold (Ct) less than 40 were considered positive and confirmed by qPCR analysis.

**Table 1:** Details of the PCR primers and probes used in this study.

Organisms	Gene	Primers and Probes	Sequences (5′-3′)
*Plasmodium falciparum*	*18S*	Pfalci_F	TAGCATATATTAAAATTGTTGCAG
*Plasmodium falciparum*		Pfalci_R	GTTATTCCATGCTGTAGTATTCA
*Plasmodium falciparum*		Probe	6FAM- CGGGTAGTCATGATTGAGTTCATTC
*Plasmodium malariae*	*18S*	Pmal_F	TAGCATATATTAAAATTGTTGCAG
*Plasmodium malariae*		Pmal_R	GTTATTCCATGCTGTAGTATTCA
*Plasmodium malariae*		Probe	6FAM- TTGCATGGGAATTTTGTTACTTTGAGT
*Plasmodium ovale*	*18S*	Pova_F	TAGCATATATTAAAATTGTTGCAG
*Plasmodium ovale*		Pova_R	GTTATTCCATGCTGTAGTATTCA
*Plasmodium ovale*		Probe	6VIC- TGCATTCCTTATGCAAAATGTGTTC
*Plasmodium vivax*	*18S*	Pviva_F	TAGCATATATTAAAATTGTTGCAG
*Plasmodium vivax*		Pviva_R	GTTATTCCATGCTGTAGTATTCA
*Plasmodium vivax*		Probe	6VIC- CGACTTTGTGCGCATTTTGC
*Plasmodium* sp.	*Cox1*	Plasmo_cox_15_F	AGGAACTCGACTGGCCTACA
*Plasmodium* sp		Plasmo_cox_16_R	CCAGCGACAGCGGTTATACT
*Plasmodium* sp		Probe	6FAM- CGAACGCTTTTAACGCCTGACATGG

^1^ 18S: 18S rRNA subunit coding gene.

COX1: cytochrome C oxidase subunit 1 coding gene.

### 2.4 Detection of intestinal parasites

Aliquots of stools cryopreserved at −80 °C in the Marseille laboratory were subjected to total DNA extraction by the semi-automated method of EZ1 Advanced XL (QIAGEN Instruments Hombrechtikon, Switzerland) with the DNA card bacteria V 1.066069118 QIAGEN and the EZ1 DNA tissue kit following the procedure described by the manufacturer. Real-time PCR was performed on DNA extracted by the thermal cyclers of CFX96TM and CFX384TM Real-Time PCR Detection Systems (BIO-RAD, Life Science, Marnes-la-Coquette, France) for the detection of 20 intestinal parasites following the procedure detailed below [34,35].

### 2.5 Bacterial 16S metabarcoding analysis

Stool samples were mechanical lysed using acid washed glass bead powder (G4649-500g Sigma) and 0.5 mm glass beads Cell rupture medium (Scientific Industries, Inc.) using a FastPrep BIO 101 instrument (Qbiogene, Strasbourg, France) at maximum speed (6.5 m/s) for 90 s. The DNA was then extracted following the procedures of two commercial kits, NucleoSpin Tissue (Macherey Nagel, Hœrdt, France) and method 5 using a deglycosylation step and purification on the EZ1 Advanced XL (Qiagen, Courtabœuf, France). The DNA extracted by these two procedures for each sample were then pooled and amplified PCR for 45 cycles, with the Kapa HiFi Hotstart ReadyMix 2x reagents (Kapa Biosystems Inc., Wilmington, MA U.S.A.) and V3_V4 primers from the surrounding conserved region with adapters (FwOvAd_341F TCGTCGG-CAGCGTCAGATGTGTATAAGAGACAGCCTACGGGNGGCWGCAG; RevOvAd_785R GTCTCGTGGCTCGGAGATGTGTATAAGAGACAGGACTACHVGGGTATCTAATCC). The MiSeq system (Illumina, Inc., San Diego CA 92121, USA) with a paired end-of-sequence strategy allowed the sequencing of the 16S RNA gene of the V3-V4 hypervariable region according to the procedure used at IHU-MI platform [34]. The paired reads were filtered according to the read qualities. The raw data were configured in fastaq files for R1 and R2 reads. Analyses of the Reads were performed by the pandaseq tool and the vsearch tool. Clustering of the data was carried out by the Qiime tool. The SILVA and IHU-MI Culturomics 16S databases were queried for taxonomic assignment of operational taxonomic units (OTUs). OTUs are a standardized, operational way to handle microbial diversity in DNA sequence metabarcoding-based studies and to classify groups of organisms with similar DNA sequences. The criteria established for taxonomic assignment of OTUs were as follows: 1) presence of one or more blast hits associated with a reference sequence (100 % coverage; identity >97 % corresponds to the assignment of OTUs to the species associated with the best blast hit); 2) presence of less relevant blast hits (identity between 95 and 97 %: assignment to genus level; between 90 and 95 %: assignment to the family; below 90 %: assignment to the kingdom) with, in each case, the creation of a putative species; 3) no blast hits (creation of putative new bacterial species). The analysis protocol was carried out by a bioinformatics company XEGEN [36].

### 2.6 Fungal ITS1 and ITS2 metabarcoding

The semi-automatic extraction protocol of EZ1 Advanced XL (QIAGEN Instruments Hombrechtikon, Switzerland) with the DNA card bacteria V 1.066069118 QIAGEN and the EZ1 DNA tissue kit was used to extract the total DNA as detailed here [34]. The amplification reaction mix consisted of 12.5 μl AmpliTaq Gold master mix, 0.75 μl of each primer (Eurogentec, Seraing, Belgium), 6 μl distilled water and 5 μl DNA template for 25 μl volume. The amplification programme was as follows: 95 °C for 10 min, 95 °C for 30 s, (55 °C or 52 °C) for 30 s, 72 °C for 1 min, and 72 °C for 5 min. Amplification of the ITS1 and ITS2 region using the primers described herein [32] with independent hybridisation temperatures of 52 °C and 55 °C, were made in triplicate. The amplicons of the replicated PCRs and the two hybridisation temperatures relating to ITS1 and ITS2 were pooled for metabarcoding on the MiSeq platform.

After purification on AMPure beads (Beckman Coulter Inc., Fullerton, CA, USA), concentration was measured using high sensitivity Qubit technology (Beckman Coulter Inc., Fullerton, CA, USA) and dilution to 3.5 ng/μl was performed. At this step, Illumina sequencing adapters and dual-index barcodes were added to the amplicon. After purification on AMPure beads (Beckman Coulter Inc., Fullerton, CA, USA), this library was pooled with 94 other multiplexed samples. The global concentration was quantified by a Qubit assay with the high sensitivity kit (Life technologies, Carlsbad, CA, USA). Before loading for sequencing on MiSeq (Illumina Inc., San Diego, CA, USA) the pool was diluted at 8pM. Automated cluster generation and paired-end sequencing with dual index reads was performed in a single 39-h run in a 2x250bp. The paired reads were filtered according to the read qualities. The raw data were configured in fastaq files for R1 and R2 reads.

The Illumina MiSeq sequences analysis was performed by PIPITS, an automated pipeline for the analysis of fungal ITS (internal transcribed spacer) sequences from the Illumina sequencing platform, hereafter referred to as the protocol [37]. The pipeline consists in the following consecutive steps: 1) preparation of raw sequences (joining, conversion, quality filtering, re-labelling and file formatting), 2) extraction of ITS fungi and read re-orientation, 3) processing of the reads to produce an OTU abundance table and taxonomic assignment table for downstream analysis. In this case, the extracted ITS2 and ITS1 sequences were analysed for the processes to obtain the OTU table. The OTU sequences were defined as a cluster of 97 % sequence identity. The last step generated the repseqs.fasta file representing the OTU sequences. These OTU sequences were manually queried via BLASTN against the nucleotide NCBI with the search parameters: 1) rRNA genes internal transcribed spacer region (ITS) from fungi type and reference material and, if the first query yielded <97 % identity, 2) the nucleotide collection (nt) to improve the taxonomic assignment that was generated by PIPITS. The taxon selection criteria were defined as follows: PID (percentage of identity) > 97 % assignment to species; PID between 95 and 97 %: assignment to genus level; PID between 90 and 95 %: assignment to the family; PID below 90 %: assignment to the kingdom.

### 2.7 Statistical analysis

The covariates were described via median, interquartile range, mean, and standard deviation computed with the GraphPad Prism ver. 5.03 for Windows software. Two malaria phenotypes were analysed: 1) the children who developed at least one malaria attack were compared to those who developed no malaria attack, and 2) the children who developed at least one asymptomatic *Plasmodium* parasitemia episode, were compared to those who developed no *Plasmodium* parasitemia episode during the 16 months of follow-up. Because several studies have indicated that Fulani people were less susceptible to malaria than Dogon people [4,5] we tested whether malaria susceptibility/resistance differed between the children of these two sympatric ethnic groups. A logistic regression model of SPSS 12.0 for Windows was used to assess the relationship between gut bacteria and fungi community structure and malaria infection by adjusting for age, gender, ethnicity, and the presence of eukaryotic enteric pathogens. In this analysis, the children with asymptomatic *Plasmodium* parasitemia at baseline, were excluded. The following transformations: square-root, square, and natural logarithm of the continuous variables, including age and alpha diversity indices of the bacterial and fungal communities, were tested and the best-fitting transformation (assessed via Akaike Information Criterion) of each was used in the logistical regression model. The logrank (Mantel-Cox) test was used to compare the survival distributions between groups with GraphPad Prism. The PAST4 software (PAleontological STatistics Version 4.01) was used to compare groups in terms of diversity (Shannon and Simpson indices) and richness (Chao-1 indices, number of observed OTUs) of bacterial and fungal species, and to explore the beta diversity between children with distinct malaria phenotypes via a Principal coordinate analysis (PCoA) graph and the non-parametric PERMANOVA (Permutational Multivariate Analysis of Variance). The comparison of the microbiota community between groups was carried out using the Linear Size Effect Discriminant Analysis (LDA LEfSe) available at http://huttenhower.sph.harvard.edu/galaxy/. All statistical tests were two-tailed; statistical significance threshold was set at P < 0.05.

## 3 Results

### 3.1 Children's baseline characteristics

Three hundred (300) children were included in the cohort study; the median age of the cohort population was 8 IQR [7,8], respectively, from which the ages were stratified. Stools were able to collect from 296/300 children. Microbiota analysis of the faeces of the 296 children was performed. From these apparently healthy 296 children, asymptomatic *Plasmodium* sp. parasitemia was detected by PCR in 35 (12 %). Asymptomatic baseline *P. falciparum* parasitemia was 11 % (33/296)) or *P. ovale* 0.6 % (2/296). From eukaryotic enteric pathogens protists, including *Blastocystis* sp. (49.7 %) and *Giardia lamblia* (29 %) were the more prevalent, whereas helminths were rarely detected, i.e., *Trichuris trichiura* was present in only 1 % and *Schistosoma mansoni* in 0.3 % (Table 2). The number of malaria attacks totalled 107 and the number of children carriage of asymptomatic *Plasmodium* parasitemia was 82 during the 16 months of follow-up. Age group 0-4-year-old had fewer malaria attacks and asymptomatic *Plasmodium* parasitemia compared to the older age group (Table S1).

**Table 2:** Baseline characteristics of the children included in the cohort study, by age group.

Characteristics	Age groups				Total
	6 mo-4 y	5-8 y	9-11 y	12-15 y	
**N (%)**	78 (26 %)	90 (30 %)	69 (23 %)	63 (21 %)	(n = 300)
**Male**	37 (47 %)	48 (53 %)	33 (47 %)	28 (44 %)	146 (47 %)
**Ethnicity**					
**Dogon**	55 (70 %)	61 (68 %)	46 (67 %)	38 (60 %)	200 (66 %)
**Songhai**	7 (9 %)	6 (6.7 %)	7 (10 %)	8 (13 %)	28 (9 %)
**Fulani**	2 (2.6 %)	5 (5.6 %)	4 (5.8 %)	6 (9 %)	17 (6 %)
**Others**	14 (18 %)	18 (20 %)	12 (17 %)	11 (17 %)	55 (18 %)
**Malaria attack**	0	0	0	0	0
**Subjected to qPCR (N)**	77	90	67	62	296
***Plasmodium* sp.**	2 (2.6 %)	8 (9 %)	8 (12 %)	17 (27 %)	35 (12 %)
***P. falciparum***	2 (2.6 %)	8 (9 %)	7 (10 %)	16 (25 %)	33 (11 %)
***P. ovale***	0	0/90	0/67	2 (3.2 %)	2 (0.6 %)
***Blastocystis* sp.**	26 (34 %)	49 (54 %)	41 (61 %)	31 (50 %)	147 (49.7 %)
***Giardia lamblia***	20 (26 %)	34 (38 %)	21 (31 %)	12 (19 %)	87 (29 %)
***Dientamoeba fragilis***	0	1 (1 %)	2 (3 %)	0	3 (1 %)
***Balantidium coli***	0	1 (1 %)	0	0	1 (0.3 %)
***Encephalitozoon intestinalis***	0	0	0	0	0
***Cyclospora cayetanensis***	0	1 (1 %)	0	0	0
***Cryptosporidium parvum***	0	0	0	0	0
***Entamoeba histolytica***	0	1 (1 %)	1 (1.4 %)	2 (3.2 %)	4 (1.3 %)
***Isospora belli***	0	0	1 (1.4 %)	0	1 (0.3 %)
***Enterocytozoon bieneusi***	7 (9 %)	0	0	3 (4.8 %)	10 (3.4 %)
***Ancylostoma duodenale***	0	0	0	0	0
***Ascaris lumbricoides***	0	0	0	0	0
***Hymenolepis diminuta***	0	0	0	0	0
***Necator americanus***	0	0	0	0	0
***Schistosoma mansoni***	0	0	0	1 (1.6 %)	1 (0.3 %)
***Strongyloides stercoralis***	0	0	0	0	0
***Taenia solium***	0	0	0	0	0
***Taenia saginata***	0	0	0	0	0
***Trichuris trichiura***	0	1 (1 %)	0	2 (3.2 %)	3 (1 %)
***Enterobius vermicularis***	0	0	0	0	0

### 3.2 Gut bacteria 16S metabarcoding

**Fig. 1:**
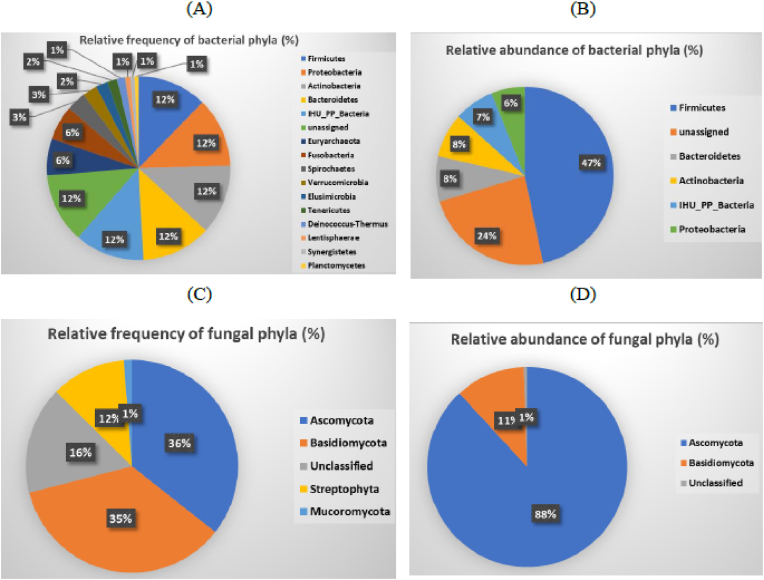
Relative frequency (A) and abundance (B) of the major gut bacterial phyla, and relative frequency (C) and abundance (D) of the major gut fungal phyla, characterised via 16S or ITS metabarcoding.

From the 296 stool samples, 24, 913, 960 sequences were generated for taxonomic assignment. Sequences not classified at the species level and the other classes were indicated “unassigned” for taxonomy. Some classified sequences were also labelled as IHU_Bacteria (bacteria from the IHU database) with distinct numbers. The analysis of OTUs in the children's gut bacterial communities showed a relatively evenly distributed frequency of the major phyla, Firmicutes (12 %), Bacteroides (12 %), Actinobacteria (12 %) and Proteobacteria (12 %), and a relative higher abundance of Firmicutes (47 %) compared to other phyla, including Bacteroides (8 %), Actinobacteria (8 %) and Proteobacteria (6 %) (Fig. 1A and B).

### 3.3 Gut fungi ITS metabarcoding

The ITS1 region analysis yielded 647,816 reads and 532 single OTUs; the ITS2 region yielded 1,975,320 reads and 479 single OTUs. Of the 1011 detected fungal taxa, 53 were identified in ITS1 reads and 479 in both ITS1 and ITS2 reads. The ITS1 and ITS2 OTU tables were combined for all further analyses.

**Fig. 2:**
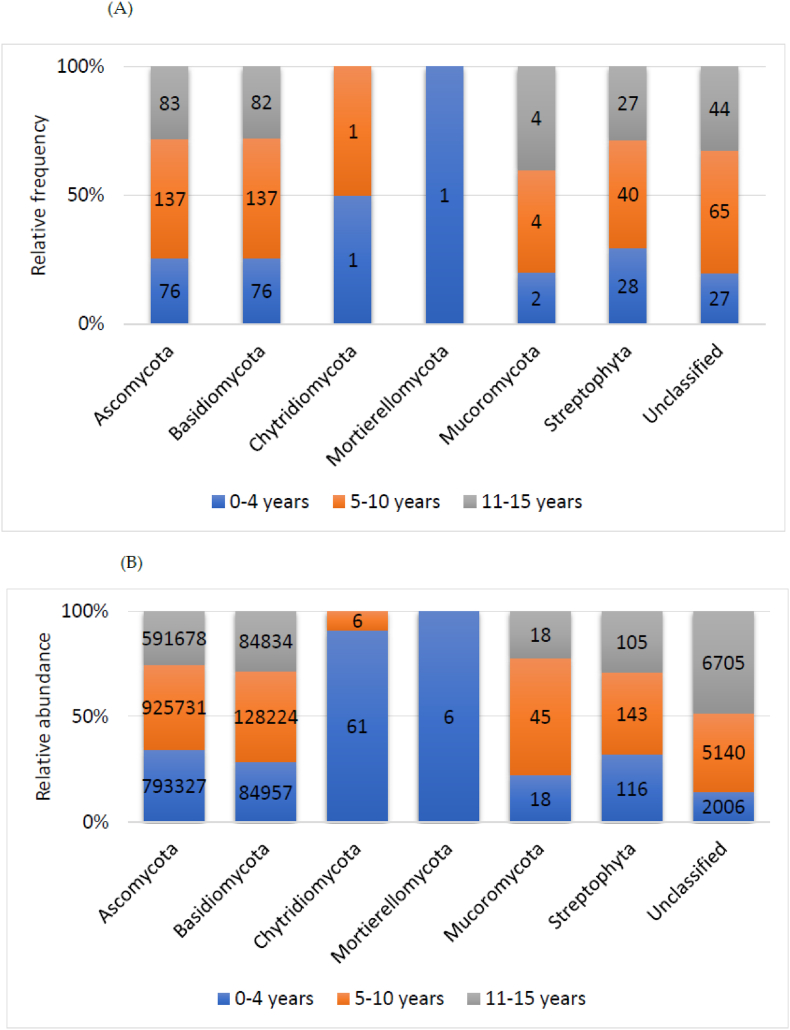
Distribution of the relative frequency A), and abundance B) of gut fungi phyla metabarcoding reads according to age. (Figures inside the bars are the mean abundance and number of the phylum for each group).

The analysis of OTUs in the children's gut fungal communities showed that relatively evenly distributed frequency of the two major phyla, Ascomycota (35.5 %) and Basidiomycota (35.3 %). It should be noted that Plantae Streptophyta accounted for 11 % of the OTUs (mostly amplified with the ITS2 barcode) and 16.3 % remained unclassified. The most abundant phyla were Ascomycota (88 %) and Basidiomycota (11 %) (Fig. 1C and D). Because gut fungi are less complex and relatively less known than bacterial communities, we further analysed the distribution of the fungal taxa according to the children's age groups. All fungi phyla were detected in children under the age of five; each phylum frequency was relatively higher in the 5 to 10-year-old group (Fig. 2A). Fungal phyla abundance relatively peaked in children under the age of 5 to 10-year-old and then decreased with age (Fig. 2B).

### 3.4 Malaria disease patterns during the 16 months of follow-up

Thirty-five (35) children with asymptomatic *Plasmodium* parasitemia at baseline were excluded from these analyses and the median age of the remaining 258 children was seven years (range = 0–15, IQR [4–10.25]). A natural logarithmic transformation of age (in years) was best suited to the logistical regression model of both malaria attacks and asymptomatic *Plasmodium* parasitemia risks. The best-fitting multivariate logistical regression analysis model found that age (OR = 1.66 (1.11–2.48), p = 0.014), and OTU richness (OR = 1 (1.000–1.002), p = 0.004), were independently statistically significantly associated with the risk of developing at least one attack (Table 3).

**Table 3:** Logistic regression analysis of the association between age and gut bacterial and fungal community structure with the risk of malaria attack.

Variables	Univariate analysis		Multivariate analysis	
	Odd Ratio (95 %CI)	P value	Odd Ratio (95 %CI)	P value
Age	6.32 (1.57–25.35)	0.009	1.66 (1.11–2.48)	0.014
**Bacteria community**	(n = 107/300, 35.67 %)			
OTU Richness	1.001 (1–1.002)	0.001	1 (1–1.002)	0.004
Chao-1 index	2.04 (1.05–3.94)	0.035	1 (1–1.002)	0.432
Shannon index	1.51 (0.81–2.83)	0.197	–	–
Simpson index	0.66 (0–7569.24)	0.929	–	–
**Fungi community**	(n = 107/300, 35.67 %)		–	–
OTU Richness	1 (0.994–1)	0.647	–	–
Chao-1 index	0.998 (0.989–1.007)	0.633	–	–
Shannon index	0.83 (0.40–1.76)	0.623	–	–

Best-fitting transformation model used natural logarithm function of: Age; Chao indices; Richness; and Simpson indices; and power function of Shannon indices.

Similarly, age (OR = 2.10 (1.32–3.35), p = 0.002), and the diversity indices, Shannon H (OR = 5.34 (1.05–27.67), p = 0.043) and the Simpson D natural logarithm (OR = 0.001 (0–0.026), p = 0.024), were independently statistically significantly associated with the risk of developing at least one asymptomatic *Plasmodium* parasitemia episode (Table S2).

Both age and OTU richness were significantly associated with both malaria attack and asymptomatic *Plasmodium* parasitemia in the univariate analysis. The survival analysis showed that being over the age of four was associated with an increased risk of both malaria attack (p = 0.015) and asymptomatic *Plasmodium* parasitemia (p < 10-3) (Figs. S1A and S1B). The median OTU richness was 1106 (112–2551) and the median Chao-1 index was 1303 (428–3979). To estimate the risk of malarial infection, the cohort of 258 children were divided in two groups either lesser than or equal to and above the median of the richness indices (observed OTU richness and Chao-1). Cox regression analysis showed that a relatively low OTU richness was associated with a significantly lower risk of malaria attack than with high OTU richness in children (Hazard Ratio = 0.59; 95 %CI [0.42–0.84], p = 0.0031). Similarly, children with a relatively low OTU richness showed a lower risk of asymptomatic *Plasmodium* parasitemia episodes than children with higher OTU richness (Hazard Ratio = 0.60; 95 %CI [0.44–0.81], p = 0.0009) (Figs. S2A and S2B). To assess whether age could be a confounding factor, the same analysis was conducted in children aged 0–4 years old or aged 5 years old and above. Children aged 0–4 years old with an OTU richness below the median value (1109; IQR [680–1307]) showed a lower risk of both malaria attack and asymptomatic *Plasmodium* parasitemia episodes than children with an OTU richness above the median value (p = 0.03) (Fig. S3). This effect was even enhanced in children aged 5–15 years, where those with an OTU richness below the median value (1109; IQR [680–1307]) showed a lower risk of both malaria attack and asymptomatic *Plasmodium* parasitemia episodes than those with an OTU richness above the median value (p < 0.0001) (Fig. S4).

### 3.5 Malaria in the Dogon and Fulani ethnic groups

Regarding these two sympatric ethnic groups in Bandiagara, more Dogon (n = 200) than Fulani (n = 17) children were included in this study. The median age of the Dogon (7 [[4], [5], [6], [7], [8], [9], [10], [11], [12], [13], [14], [15]]) and Fulani (10 [[1], [2], [3], [4], [5], [6], [7], [8], [9], [10], [11], [12], [13], [14]]) children was homogeneous (p = 0.12). Overall, 74 (37 %) of the Dogon and four (24 %) of the Fulani children (p = 0.306) developed at least one malaria attack and 53 (27 %) of the Dogon and seven (41 %) and of Fulani children (p = 0.257) developed at least one asymptomatic *Plasmodium* parasitemia episode during the 16-month follow-up period. Because of the relatively small number of Fulani enrolled into the study, the impact of the gut bacterial and fungal communities in these ethnic groups was not investigated further.

### 3.6 Gut bacterial community structure associated with malarial risk

The association of age, sex, eukaryotic enteric pathogens, and gut bacterial and fungal community structures with either the risk of a malaria attack or asymptomatic *Plasmodium* parasitemia, were assessed using an unconditional logistic regression analysis. Univariate analysis showed that age and gut bacteria richness indices (Table 3) were significantly associated with malaria attacks (p < 0.05). In the multivariate analysis, age and OTU richness were statistically significantly associated with malaria attacks (OR = 2.62, 95 %CI [1.24–5.54] and OR = 1.00 (95 %CI [1.00–1.002]) respectively (Table 3). Similarly, age (OR = 2.10, 95 %CI [1.32–3.35]) was statistically significantly associated with asymptomatic *Plasmodium* parasitemia (Table S2).

**Fig. 3:**
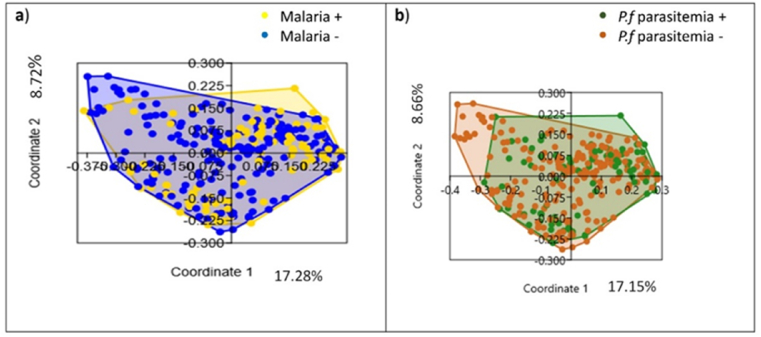
Gut bacterial community structure and malaria risk. Principal Coordinates Analysis (PCoA) of the gut bacterial community a) in children who experienced (yellow dots), or did not experience (blue dots), at least one malaria attack within 16 months of follow-up (Permanova test, p = 0.0054); b) in children who developed (green dots), or did not develop (pink dots), at least one asymptomatic *Plasmodium* parasitaemia episode within 16 months of follow-up (Permanova test, p = 0.012).

The gut bacterial community structure of children who developed at least one malaria attack were compared with those who did not, and that of those who developed at least one asymptomatic *Plasmodium* parasitemia episode was compared with those who did not. At the phylum level, the relative abundance of the main bacterial phyla were Firmicutes (53.95 %), Proteobacteria (53.84 %), Actinobacteria (52.9 %), and Bacteroides (49.54 %) in the children who developed at least one malaria attack compared to those who did not (Fig. S5); and Proteobacteria (55.3 %), Firmicutes (52.83 %), Actinobacteria (49.97 %), and Bacteroides (49.52 %) in children who developed at least one asymptomatic *Plasmodium* parasitemia episode compared to those who did not (Fig. S6). The impact of the gut bacteria community structure on malarial risk was further assessed via Principal Coordinates Analysis (PCoA) and tested using PERMANOVA, based on the Bray-Curtis similarity measure. It significantly differed in the children who developed at least one malaria attacks compared to those who did not (p = 0.005) (Fig. 3a) and differed in those who developed at least one asymptomatic *Plasmodium* parasitemia episode compared to those who did not (p = 0.012) (Fig. 3b).

**Fig. 4:**
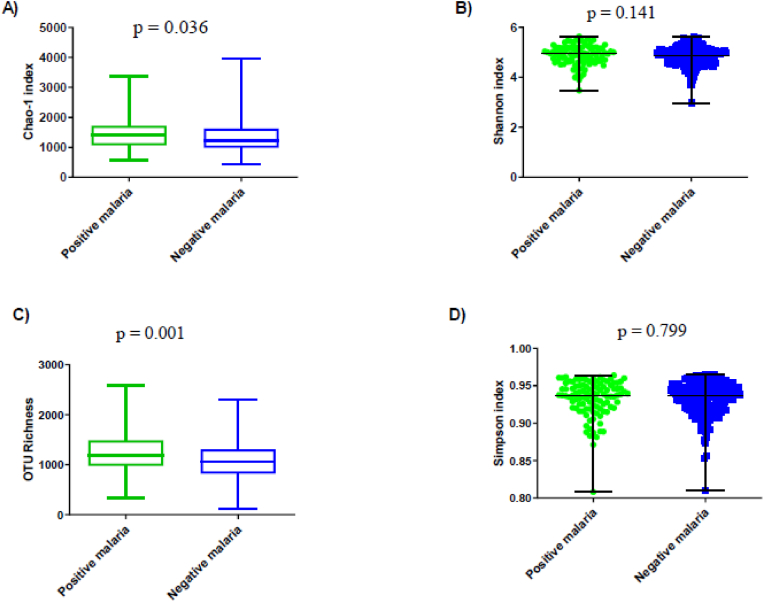
Richness and diversity indices of the gut bacterial community in children who experienced or did not experience at least one malaria attack within 16 months of follow-up. Scatter dot plot and Box-Whisker's graph shows Median, Min and Max of children who experienced at least one malaria attack (green) compared to control (blue) A) for chao-1 index (p = 0.036); B) for Shannon index (p = 0.141); C) for OTU Richness (p = 0.001); D) for Simpson index (p = 0.799).

Bacterial OTU richness (Chao-1 index and observed OTU richness) and diversity in Shannon H and Simpson D indices were evaluated according to malaria status and age (Table S3). The diversity indices were homogeneously distributed in each of the groups of children (Table S3). In contrast, the Chao-1 index (median 1457 [1352–1561]) in the children who developed at least one malaria attack was significantly higher than in those who did not (1330 [1257–1402]) (p = 0.036). Also, the median bacterial OTU richness observed (1233 [1152–1313]) in the children who developed at least one malarial attack was statistically significantly higher (p = 0.001) than that observed in those who did not (1076 [1022–1129]) (Fig. 4; Table S4).

**Fig. 5:**
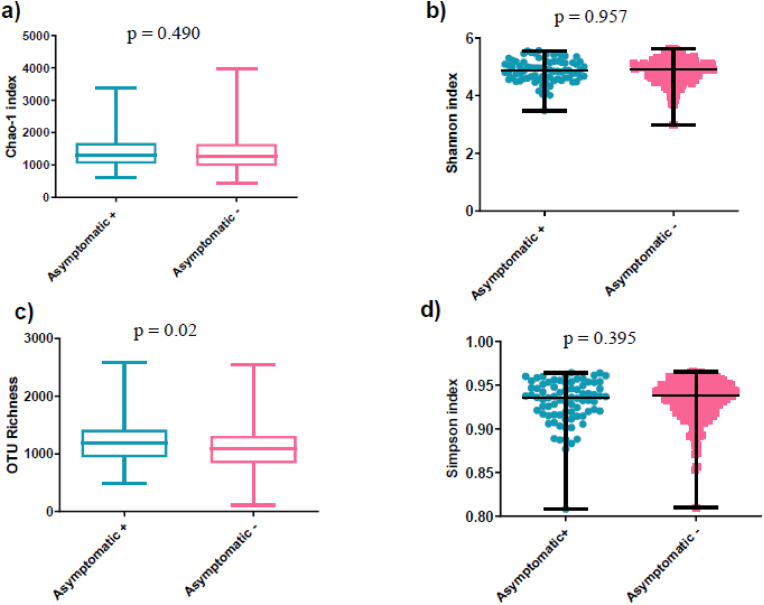
Richness and diversity indices of gut bacterial community of children who developed or did not develop at least one asymptomatic *Plasmodium* parasitaemia episode within 16 months of follow-up. Median and range of chao-1 index (p = 0.049) a); of Shannon index (p = 0.957) b); for OTU richness (p = 0.02) c) and Simpson index (p = 0.395) d) of children who developed at least one asymptomatic *Plasmodium* parasitaemia episode (light blue) compared to control (pink) for Scatter dot plot, Box and Whiskers graph.

We found that the median bacterial OTU richness observed in the children who developed at least one asymptomatic *Plasmodium* parasitemia episode (1217 [1130–1305]) was higher (p = 0.02) than in those who did not (1099 [1046–, 1151]) (Fig. 5; Table S5).

**Fig. 6:**
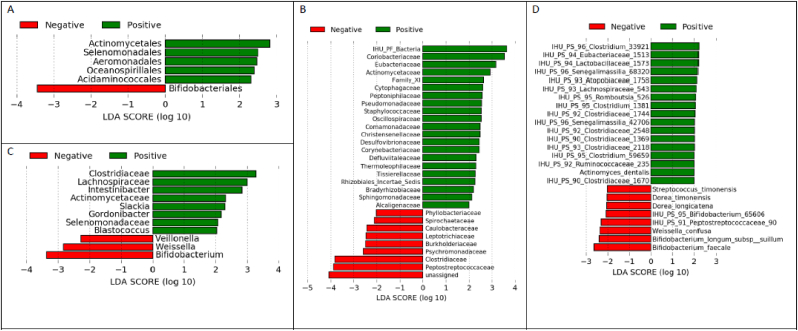
Linear size effect discriminant analysis (LDA LEfSe) of the gut bacterial community structure at, A) Order, B) Family, C) Genus and D) species levels, between children who did (green), or did not (red), experienced at least one malaria attack within 16 months of follow-up. Horizontal bars represent the effect size for each taxon. The length of the bar represents the log10 transformed LDA score, indicated by vertical dotted lines.

The presence of significantly differentially distributed bacterial taxa in children who developed at least one malaria attack and those who did no was assessed using a linear size effect discriminant analysis (LDA LEfSe) at various taxonomical levels (Fig. 6AD). At the Order level, Actinomycetales, Selenomonadales, Aeromonadales, Oceanospirillales, and Acidaminococcales were more abundant in the children who developed at least one malaria attack, whereas Bifidobacteriales were more abundant in those who did not. At the Family level, mainly Coriobacteriaceae, Eubacteriaceae, Actinomycetaceae, and Staphylococacceaea were more abundant in the children who developed at least one malaria attack, whereas Peptostreptococcaceae, Clostridiaceae, Psychromonadaceae, and Burkholderiaceae were more abundant in those who did not. At the genus level, IHU-Clostridiacea, IHU-Lachnospiraceae, Intestinibacter, IHU-Actinomycetaceae, Slackia, Gordonibacter, IHU-Selenomonadaceae and Blastococcus were more abundant in the children who developed at least one malaria attack, whereas *Bifidobacterium*, *Weissella*, and *Veillonella* were more abundant among those who did not. At the species level, *Clostridium* sp. were most abundant in the children who developed at least one malaria attack, whereas *Bifidobacterium faecale*, *Bifidobacterium longum* subsp. *suillum*, *Weissela confusa*, Peptostreptococcaceae sp., *Dorea longicatena*, *Dorea timonensis*, and *Streptococcus timonensis*, were more abundant in those who did not (Fig. 6A–D).

Regarding the effect of age on bacterial community structure: in 0–4 years old children who developed at least one malaria attack, from 68 species, Terrisporobacter_petrolearius, UHI_PS_93_Ruminococcaceae_2772 were more abundant, whereas in children who did not 17 species, including *Streptococcus salivarius* subsp. *salivarius* and *Bifidobacterium faecale* were more abundant (Fig. S7); in 5–15 years old children who developed at least one malaria attack, 14 species, including *Streptococcus lutetiensis* and IHU_PS_95_Gemmiger_1543, were more abundant, whereas 9 species, including *Prevotella copri* and IHU_PS_96_Roseburia_2390, were more abundant in children who did not (Fig. S8).

**Fig. 7:**
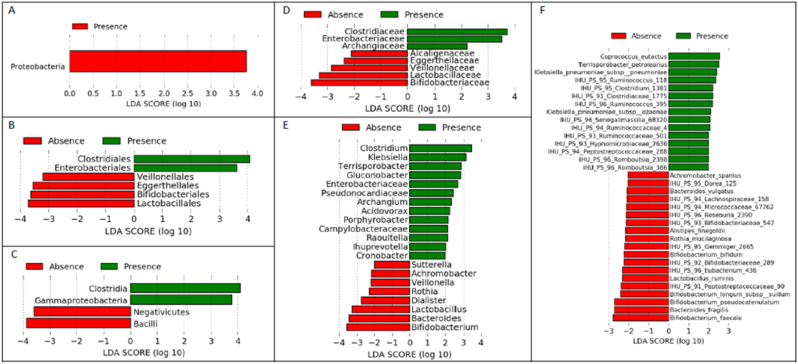
Linear size effect discriminant analysis (LDA LEfSe) between of bacterial gut microbiota of children who developed (green) or did not develop (red bars) at least one asymptomatic *Plasmodium* parasitaemia episode within 16 months of follow-up at the level of taxonomic classes of the bacterial community structure at, A) Phyla, B) Order, C) Class, D) Family, E) genus, and F) species levels. Horizontal bars represent the effect size for each taxon. The length of the bar represents the log10 transformed LDA score, indicated by vertical dotted lines.

Regarding the children who developed at least one asymptomatic *Plasmodium* parasitemia episode, the LDA LEfSe test showed a higher abundance of the Proteobacteria phylum, whereas Bacilli and Negativicutes were more abundant in children who developed no parasitemia. At the class level, Gammaproteobacteria and clostridia were more abundant in children who developed at least one asymptomatic episode of *Plasmodium* parasitemia and Bacilli and Negativicutes were more abundant in children who did not develop at least one asymptomatic *Plasmodium* parasitemia. At the order level, children who developed at least one asymptomatic episode of *Plasmodium* parasitemia had a greater abundance of Clostridiales and Enterobacteriales, while children without an asymptomatic episode of *Plasmodium* parasitemia showed a greater abundance of Lactobacilli, Bifidobacteriales, Eggerthellales and Veillonellales. The same trends were observed at the Family level: Clostridiaceae, Enterobacteriaceae, Archandiaceae were more abundant in the children who developed at least one asymptomatic *Plasmodium* parasitemia episode, whereas Lactobacillaceae, Bifidobacteriaceae, Eggerthellaceae, and Veillonellaceae were more abundant in those who did not. At the genus level, *Clostridium*, *Klebsiella*, and IHU-Enterobacteriaceae were more abundant in the children who developed at least one asymptomatic *Plasmodium* parasitemia episode, whereas *Bifidobacterium*, *Bacteroides*, *Lactobacillus*, *Dialister*, *Rothia*, and *Veillonella* were more abundant in those who did not. At species level, *Clostridium* sp., *Klebsiella* sp., *Ruminococcus* sp., *Ramboutsia* sp. were more abundant in children who developed at least one asymptomatic *Plasmodium* parasitemia episode, whereas *Bifidobacterium* sp., *Bacteroides fragilis*, and *Lactobacillus ruminis* were more abundant in those who did not (Fig. 7A–D).

Regarding the effect of age on bacterial community structure; children from 0 to 4 years old who developed at least one asymptomatic episode of *Plasmodium* parasitemia, *Romboutsia timonensis* and *Ruminococcus bromii* were more abundant among 107 species, whereas in children who did not 5 species, *Collinsella aerofaciens* and *Bifidobacterium longum* subsp. *longum* were more abundant (Fig. S9); in 5–15 years old group who developed at least one asymptomatic episode of *Plasmodium* parasitemia, 6 species, including *Klebsiella pneumoniae* subsp. *pneumoniae* and IHU_PS_96_*Ruminococcus*_395, were more abundant, whereas 10 species, including *Bacteroides fragilis* and IHU_PS_96_*Eubacterium*_436, were more abundant in children who did not (Fig. S10).

### 3.7 Gut fungal community structure associated with malaria risk

**Fig. 8:**
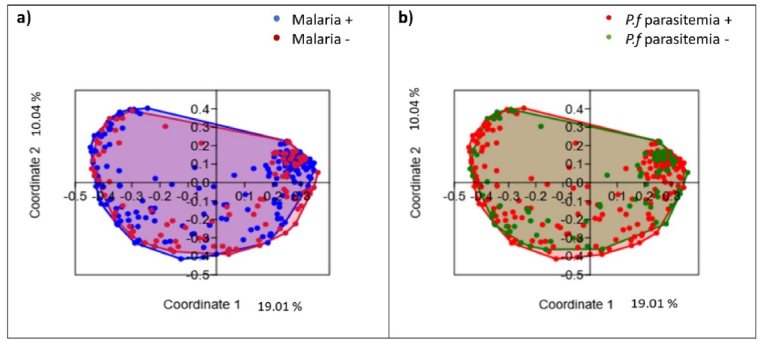
Gut fungal community and malaria risk. Principal Coordinates Analysis (PCoA) of the gut fungal community structure a) in children who experienced at least one malaria attack (crimson dots) or those who did not (blue dots) (PERMANOVA test, p = 0.27); b) in children who developed at least one episode of *Plasmodium* parasitaemia (red dots) or did not (green dots) (PERMANOVA test, p = 0.76).

The alpha diversity indices did not statistically significantly differ between the children who developed at least one malaria attack (Table S4), asymptomatic *Plasmodium* parasitemia episode (Table S5) and those who did not (Fig. S11). The beta diversity of the fungal community between children with at least one malaria attack (n = 105) and children tested negative (n = 191) was analysed by Principal Coordinated Analysis (PCoA) based on the Bray-Curtis similarity index. In line with a non-statistically significant (p = 0.27) PERMANOVA test, PCoA showed no pattern (Fig. 8a). Similar results were observed between the children who developed at least one asymptomatic *Plasmodium* parasitemia episode (n = 81) and those (n = 215) who did not (PERMANOVA test, p = 0.76) (Fig. 8b).

### 3.8 Gut fungal community structure

**Fig. 9:**
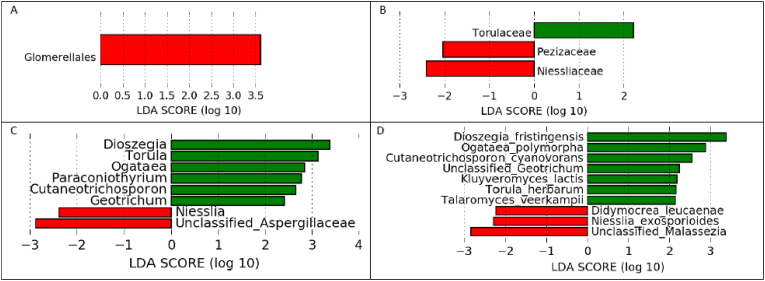
Linear size effect discriminant analysis (LDA LEfSe) of the fungal community structure at B) family, C) genus, and D) species levels, between children who experienced (green), or did not experience (red), at least one malaria attack within 16 months of follow-up. For A) Order children who experienced at least one malaria attack within 16 months of follow-up (red) were significant. Horizontal bars represent the effect size for each taxon. The length of the bar represents the log10 transformed LDA score, indicated by vertical dotted lines.

The relative mean of phyla abundance in children who developed at least one malaria attack was Ascomycota (46 %), Basidiomycota (62 %) compared to children with no malaria attack (Fig. S12). In children who developed at least one asymptomatic *Plasmodium* parasitemia episode, the relative mean of phyla abundance was Ascomycota (52 %), Basidiomycota (50 %) against children without an asymptomatic *Plasmodium* parasitemia episode (Fig. S13). At the phyla and class level, no abundance of fungi was significant between children who developed at least one attack of malaria and those who did not. However, the most significantly abundant fungi in children who developed at least one malaria attack were the orders Glomerelalles, Families Turolaceae, genera Dioszegia, Turola, Cutaneotrichosporon and Geotrichum, species *Dioszegia fristingensis*, *Ogataea polymorpha*, *Cutaneotrichosporon cyanovorans*, Unclassified_*Geotrichum*, *Kluyveromyces lactis*, *Torula herbarum* and *Talaromyces veerkampii* (Fig. 9A–D). The gut fungi of children who developed an asymptomatic episode of *Plasmodium* parasitemia was comparable on the phyla level with those who did not. Regarding children who did not develop at least one attack of malaria, the Pezizaceae and Niessliaceae families, the Niesslia and Unclassified Aspergillaceae genera, the *Didymocrea leucaenae*, *Niesslia exosporioides* species and Unclassified *Malassezia* were the most significantly abundant (Fig. 9: B, C,D).

**Fig. 10:**
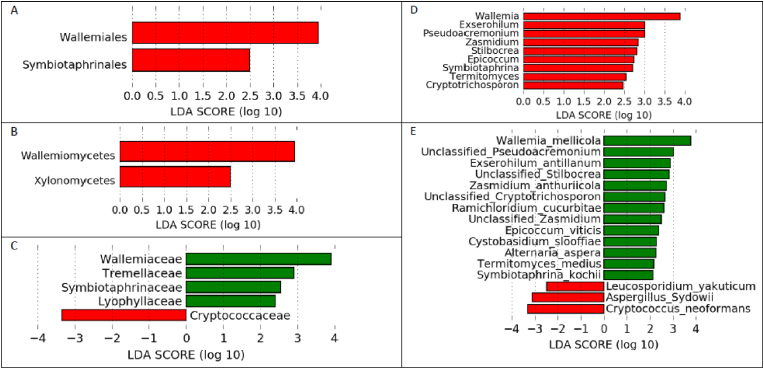
Linear size effect discriminant analysis (LDA LEfSe) of the fungi community structure at, C) Family and E) species levels, between children who developed (green), or did not develop (red), at least one asymptomatic *Plasmodium* parasitaemia episode within 16 months of follow-up. Fungi community structure at A) Order, B) Class and D) genus were significant in children who developed (red) at least one asymptomatic *Plasmodium* parasitaemia episode within 16 months of follow-up. Horizontal bars represent the effect size for each taxon. The length of the bar represents the log10 transformed LDA score, indicated by vertical dotted lines.

Considering the gut fungi of children who developed an asymptomatic episode of *Plasmodium* parasitemia, the classes Wallemiomycetes and Xylonomycetes, the Wallemial and Symbiotaphrine Orders, the families Wallemiaceae, Tremellaceae, Symbiotaphrinaceae and Lyophyllaceae, genera such as *Wallemia*, *Exserohilum* and *Pseudoacremonium*, and species such as *Wallemia mellicola*, Unclassified *Pseudoacremonium* and *Exserohilum antillanum* were the most abundant (Fig. 10A–E). The gut fungal community of children who did not develop an asymptomatic *Plasmodium* parasitemia episode was characterised by the relative abundance of the Cryptococcaceae families, and the presence of *Leucosporidium yakuticum*, *Aspergillus sydowii* and *Cryptococcus neoformans* species (Fig. 10C and E).

## 4 Discussion

The findings of this study highlight the association of the gut bacteria but not fungi, community structures and specific bacteria and fungi taxa with susceptibility/resistance to malaria in children in an endemic area in Mali. The main limitations are a) the gut microbiome was analysed only at the baseline time point of the study, and it is possible that significant unmeasured variations occurred during the 16-month follow-up period; indeed, studies have shown that the gut microbiota varies according to season, lifestyle and diet [20,21]; b) the lack of information on the diet and nutritional status of children, which influence the gut microbiota, may expose the analysis to an unmeasured bias; c) the relatively small number of Fulani children included precludes any robust analysis of the differences compare to the Dogon ethnic group, that have been highlighted in previous studies on the Dogon [5,22]. Nevertheless, we found that Bacteroidetes were more abundant in Fulani children, while Actinobacteria were more abundant in Dogon children. Similarly, a relatively high abundance of Bacteroidetes has been observed in traditional societies practicing a hunter-gatherer lifestyle [23].

The main strengths of our study are a) the relatively large number of children included b) the precise identification of malaria attacks and, more particularly, of asymptomatic *Plasmodium* parasitemia episodes through monthly qPCR testing during the 16 months of follow-up, and d) the analysis of both bacterial and fungal communities coupled with the qPCR detection of the major eukaryotic enteric pathogen to characterise the children's gut microbiomes. Regarding the epidemiology of malaria, our observation that children under five years old presented fewer malarial attacks than older children, was in line with a previous study conducted in the same locality, which found fewer malaria attacks in children under two years when compared to children aged three and four [24]. Furthermore, another study in a location in Senegal where seasonal malaria chemoprevention had not been implemented, showed that the prevalence of *Plasmodium* parasitemia was 18 % in children under five years and 25 % in those aged between five and nine years [25]. It is notable that the children included in our study were not exposed to seasonal malaria chemoprevention, which has been associated with a significant reduction of malaria incidence in children under the age of five [26,27]. However, they did use both long-lasting insecticidal nets (LLINs) and indoor residual spraying of insecticide (IRS), which might explain the observed higher malaria burden in older children [28]. Indeed, age was an independent factor that was strongly associated with both malaria attack and asymptomatic *Plasmodium* parasitemia (Table 2, S2).

When investigating the gut microbiota associated susceptibility/resistance to malaria, we showed that the risks of both malaria attack and asymptomatic *Plasmodium* parasitemia significantly increased with the increase in bacteria OTU richness (Table 2, S2). These findings were confirmed by the survival analysis which found a higher risk of both malaria attack and asymptomatic *Plasmodium* parasitemia in children with a bacteria OTU richness above the population's median value and in children over the age of five. A previous study in Mali found that the gut bacterial community structure was associated with asymptomatic *Plasmodium* parasitemia but not with malaria attacks [19]. Another study in Kenya found no significant impact on the gut bacteria community structure before and after a malaria attack treated with arthemether/lumefantrine, although the authors detected sequence variants of some taxa that these episodes might have selected [29]. Several pieces of evidence point to a significant influence of the gut bacterial community on the risk of malaria infection. In a mouse model, severe *Plasmodium* infections have altered host gut homeostasis, which may contribute to malaria-related enteric bacteraemia [17]. Conversely, the gut bacterial microbiota composition can modulate the severity of *P. yoelii* 17XNL infection in mice and malaria susceptibility/resistance was transferred to germ-free mice by transferring the cecum contents of susceptible or resistant mice [2]. Our study further highlighted bacterial taxa could be differentiated between children who were susceptible or resistant to malaria attacks. Among these taxa, Clostridiacea, Lachnospiraceae, Intestinibacter, Actinomycetaceae, Slackia, Gordonibacter, Selenomonadaceae and *Blastococcus* were associated with susceptibility, while *Bifidobacterium*, *Weissella*, *Veillonella*, and *Streptococcus timonensis* were associated with resistance to malaria attacks. Furthermore, in children who were asymptomatic for malaria infection, the Proteobacteria phylum and *Clostridium*, *Klebsiella*, and Enterobacteriaceae were associated with susceptibility, while *Bifidobacterium* and *Lactobacillus* were associated with resistance to asymptomatic *Plasmodium* parasitemia. Interestingly, known “beneficial” probiotic bacteria, such as *Bifidobacterium* and *Lactobacillus* were associated with malarial resistance [30,31].

To the best of our knowledge, our study is the first to investigate the association of the gut fungal community with the risk of malaria. In a companion paper [32] we described the core gut mycobiome of theses children and compared the gut fungal community structure of breastfed children, aged 0–2 years, with other age groups. We found that the gut fungal community structure was relatively homogenous between children who were susceptible to malaria and those who were resistant to it. This finding contrasts with previous reports of a higher abundance and diversity in the gut fungal community in patients with various diseases than in healthy subjects [33]. Likewise, gut fungal community dysbiosis has been associated with irritable bowel disease [34]. An unexpected finding was the association of the human opportunistic basidiomycete yeast, *Cryptococcus neoformans*, with resistance to *Plasmodium* parasitemia. Further studies are warranted to confirm the implication of the fungal taxa that have been found to be associated with susceptibility/resistance to malaria in this exploratory study.

## 5 Conclusions

The results of this study showed that the gut bacterial community structure, but not the fungal community, is associated with susceptibility/resistance to malaria attacks and asymptomatic *P. falciparum* malaria infection. We demonstrated that gut bacteria OTU richness was independently associated with the risk of a malaria attack. Further studies are needed to confirm these findings, which points the way towards strategies aiming to reduce malaria risk in endemic areas by modulating the gut microbiota components of at-risk populations.

## CRediT authorship contribution statement

**Aly Kodio:** Writing – original draft, Investigation, Formal analysis, Data curation, Conceptualization. **Drissa Coulibaly:** Writing – review & editing, Supervision, Project administration, Investigation. **Safiatou Doumbo:** Writing – review & editing, Supervision, Project administration, Investigation. **Salimata Konaté:** Writing – review & editing, Investigation. **Abdoulaye Kassoum Koné:** Writing – review & editing, Supervision, Investigation. **Souleymane Dama:** Writing – review & editing, Investigation. **Amadou Niangaly:** Writing – review & editing, Resources, Methodology, Investigation. **Mamadou Lamine Tall:** Writing – original draft, Software, Investigation, Formal analysis, Data curation. **Ahmed Mohamed Konaté:** Writing – original draft, Investigation. **Coralie L'Ollivier:** Writing – review & editing, Supervision, Methodology, Investigation. **A. Levasseur:** Writing – review & editing, Supervision, Software, Methodology. **Fadi Bittar:** Writing – review & editing, Supervision, Software, Resources, Methodology, Conceptualization. **Abdoulaye Djimdé:** Writing – review & editing, Validation, Project administration, Funding acquisition. **Ogobara K. Doumbo:** Supervision, Resources, Project administration, Investigation, Funding acquisition, Conceptualization. **Didier Raoult:** Writing – review & editing, Validation, Supervision, Resources, Project administration, Conceptualization. **Mahamadou Ali Thera:** Writing – review & editing, Supervision, Resources, Project administration, Investigation, Data curation, Conceptualization. **Stéphane Ranque:** Writing – review & editing, Writing – original draft, Supervision, Project administration, Methodology, Investigation, Conceptualization.

## Data availability statement

The data generated and analysed in this study are available on the website of the Institut Hospitalo-Universitaire – Méditerranée Infection (IHU- Méditerranée Infection). https://www.mediterranee-infection.com/acces-ressources/donnees-pour-articles/gut-microbiota-influences-plasmodium-falciparum-malaria-susceptibility/

## Funding

This work was supported by the French Government under the ‘Investissements d'avenir’ (Investments for the Future) programme managed by the 10.13039/501100001665Agence Nationale de la Recherche (ANR, fr: National Agency for Research), (reference: Méditerranée Infection 10-IAHU-03), the IHU-Méditerranée Infection Foundation, and the MARCAD DELTAS Africa Initiative grant DEL-15-10. The DELTAS Africa Initiative is an independent funding scheme of the African Academy of Sciences' (10.13039/100017182AAS) 10.13039/501100014163Alliance for Accelerating Excellence in Science in Africa (10.13039/501100014163AESA) and supported by the New Partnership for Africa's Development Planning and Coordinating Agency (10.13039/501100009250NEPAD Agency) with funding from the 10.13039/100010269Wellcome Trust grant 107741/A/15/Z and the UK government. The views expressed in this publication are those of the author(s) and not necessarily those of the AAS, the NEPAD Agency, the Wellcome Trust, or the UK government.

## Declaration of competing interest

The authors declare the following financial interests/personal relationships which may be considered as potential competing interests: Didier Raoult reports financial support from the French National Research Agency; he is also a member of the scientific board of Eurofins and founder of a culture media company (Culture Top). Aly Kodio reports financial support from the Foundation Mediterranée Infection. Mahamadou Ali Thera reports financial support from the MARCAD DELTAS Africa Initiative. Abdoulaye Djimde reports financial support from the Wellcome Trust.
