# Supplementary material for: Gut microbiota influences Plasmodium falciparum malaria susceptibility
Source: New Microbes New Infect. 2025 Apr 14;65:101586. doi: 10.1016/j.nmni.2025.101586 (PMC12032372; doi:10.1016/j.nmni.2025.101586)
Supplement: Multimedia component 1 [file mmc1.docx]

**Additional Figures.**

**Figure S1**. Log-rank (mantel-Cox) test comparing the risk of experiencing at least one malaria attack (A) or at least one asymptomatic *Plasmodium* parasitemia episode (B) in children ≤ 4 years-old (n=74) or > four years-old (n=184), within 16 months of follow-up. The 35 children who presented with asymptomatic *Plasmodium* parasitaemia at baseline were excluded from this analysis.

**Figure S2**. Log-rank (mantel-Cox) test comparing the risk of experiencing at least one malaria attack (A) or at least one asymptomatic *Plasmodium* parasitaemia episode (B) in children with either low (n=130) or high (n=128) gut bacteria OTU richness, within 16 months of follow-up. The 35 children who presented with asymptomatic *Plasmodium* parasitaemia at baseline were excluded from this analysis. A) Fraction of malaria attack free; B) Fraction of no asymptomatic *Plasmodium* parasitemia. Children with low (red); high gut bacteria OTU richness (green).

**Figure S3.** Risk of developing within 16 months of follow-up: a) at least one malarial attack or b) at least one episode of asymptomatic *Plasmodium* parasitemia in 0 to 4 years old children with gut bacteria OTU richness below (n=38, red line) or above (n=36, green line) the median value.

**Figure S4.** Risk of developing within 16 months of follow-up: a) at least one malarial attack or b) at least one episode of asymptomatic *Plasmodium* parasitemia in 5 to 15 years old children with gut bacteria OTU richness below (n=93, red line) or above (n=91, green line) the median value.

**Figure S5**. Gut bacterial community structure. Relative abundance of phyla in children who experienced (yellow bars) or who did not experience (blue bars) at least one malaria attack within 16 months of follow-up. (Figures inside the bars are the mean abundance of the phylum for each group).

**Figure S6**. Gut bacterial community structure. Relative abundance of phyla in children who developed (yellow bars) or did not develop (blue bars) at least one asymptomatic *Plasmodium* parasitaemia episode within 16 months of follow-up. (Figures inside the bars are the mean abundance of the phylum for each group).

**Figures S7.** Linear size effect discriminant analysis (LDA LEfSe) of the gut bacterial community structure at species levels in 0 to 4 years old children who experienced (green), or did not experience (red), at least one malaria attack within 16 months of follow-up. Vertical bars represent the effect size for each taxon. The length of the bar represents the log10 transformed LDA score, indicated by vertical dotted lines.


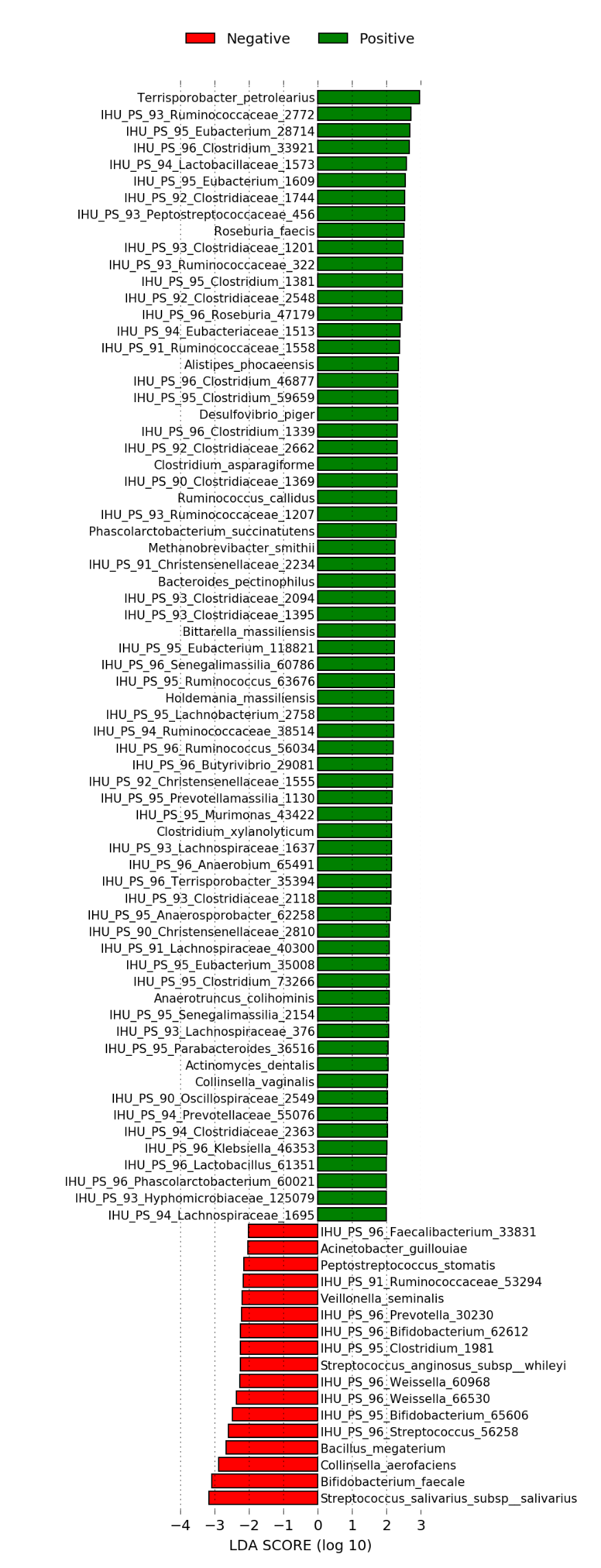


**Figure S8.** Linear size effect discriminant analysis (LDA LEfSe) of the gut bacterial community structure at species levels, in 5 to 15 years old children who experienced (green), or did not experience (red), at least one malaria attack within 16 months of follow-up. Horizontal bars represent the effect size for each taxon. The length of the bar represents the log10 transformed LDA score, indicated by vertical dotted lines.


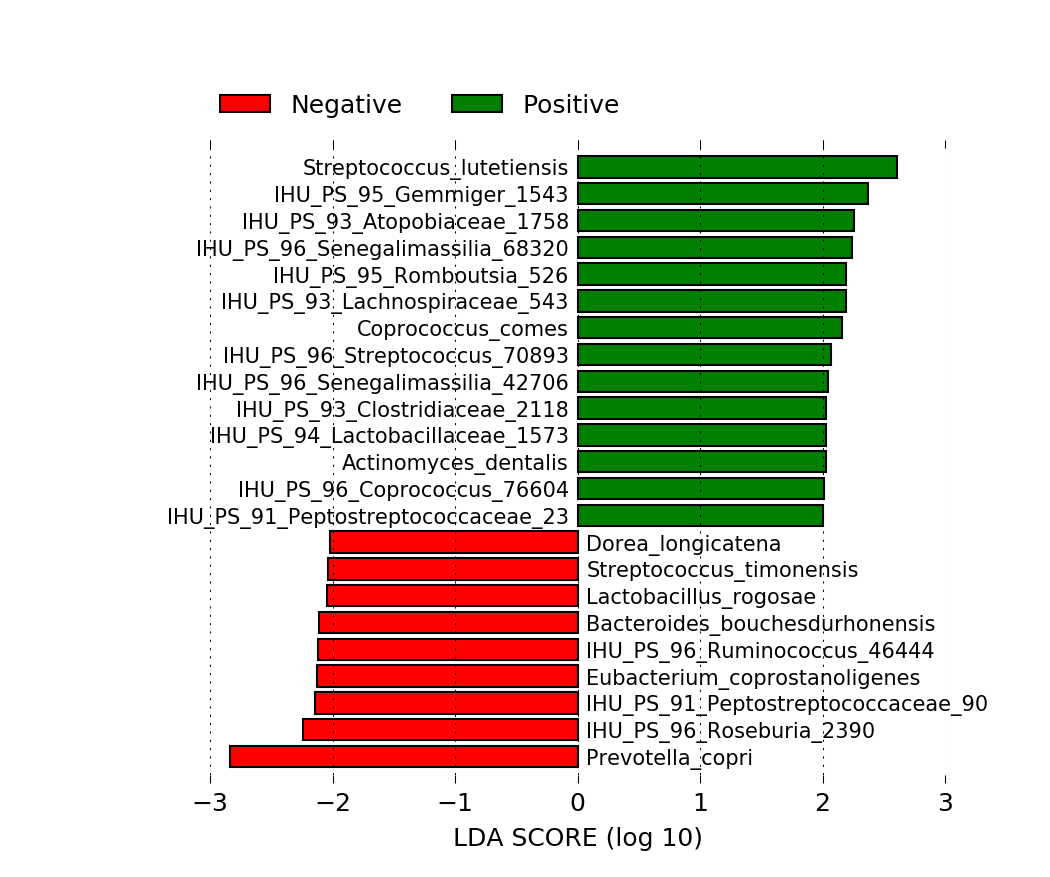


**Figure S9**. Linear size effect discriminant analysis (LDA LEfSe) of the gut bacterial community structure at species levels, in 0 to 4 years old children who developed (green), or did not develop (red), at least one asymptomatic *Plasmodium* parasitaemia episode within 16 months of follow-up. Vertical bars represent the effect size for each taxon. The length of the bar represents the log10 transformed LDA score, indicated by vertical dotted lines.


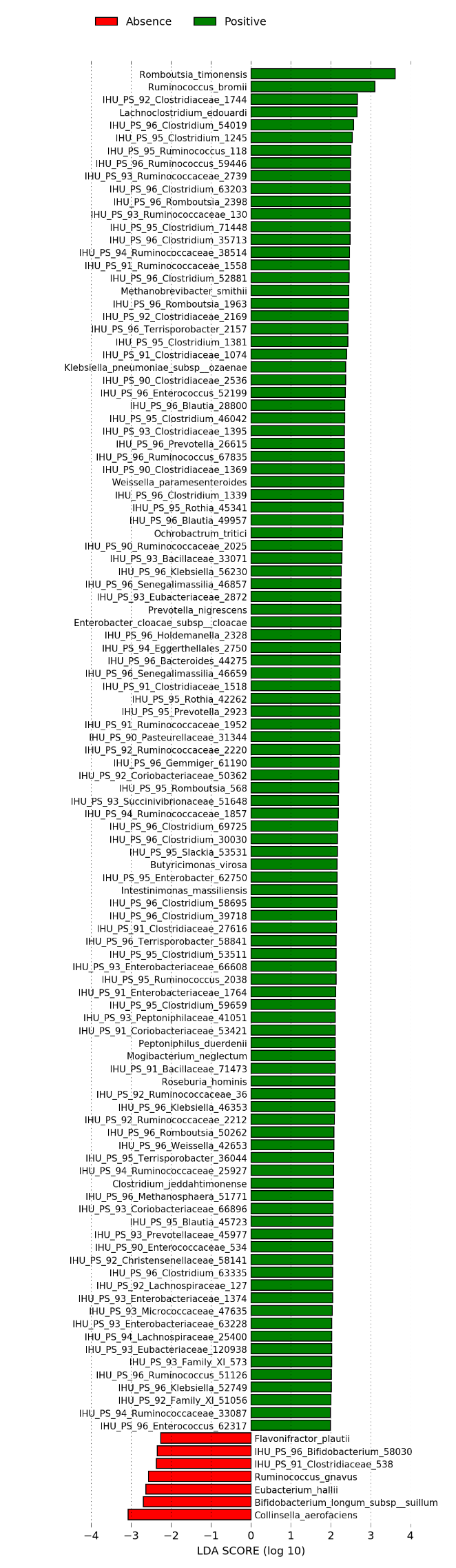


**Figure S10**. Linear size effect discriminant analysis (LDA LEfSe) of the gut bacterial community structure at species levels, in 5 to 15 years old children who developed (green), or did not develop (red), at least one asymptomatic *Plasmodium* parasitaemia episode within 16 months of follow-up. Horizontal bars represent the effect size for each taxon. The length of the bar represents the log10 transformed LDA score, indicated by vertical dotted lines.


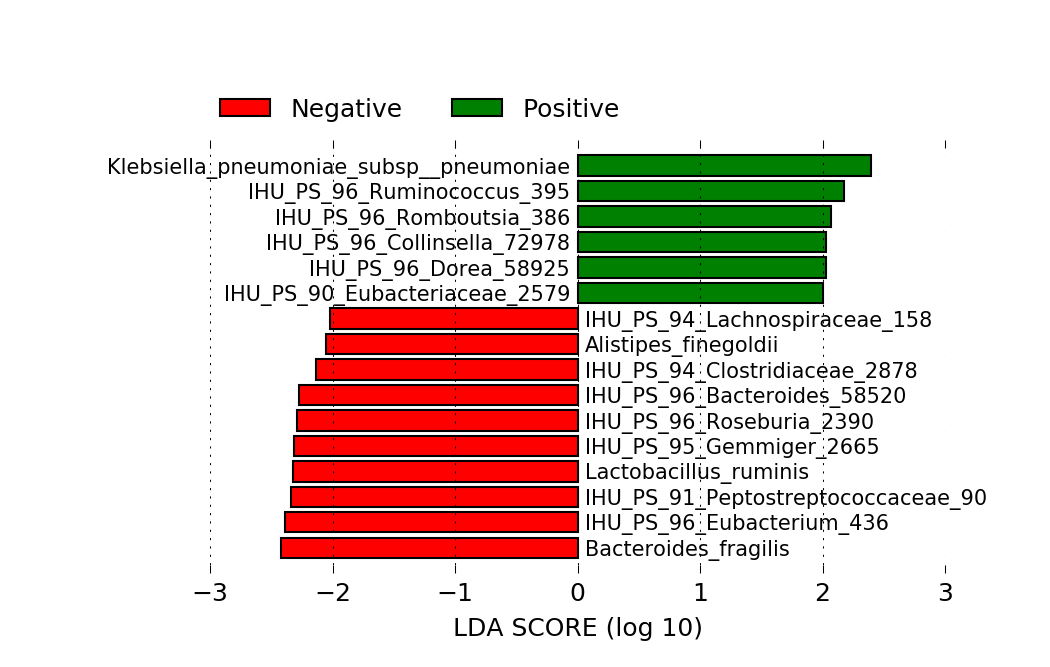


**Figure S11**. Gut fungal community structure analysis. Scatter dot plot, median and range, of the Shannon H diversity index (Man Whitney test, p=0.66) (a) and Box and Whiskers graph of the Chao-1 richness index (p=0.9) (b) in children who experienced at least one malaria attack (crimson) or who did not (blue) within 16 months of follow-up. Scatter dot plot, median and range, of the Shannon H diversity index (Man Whitney test, p=0.31) (c) and Box and Whiskers graph of the Chao-1 richness index'(p=0.87) (d) in children who developed at least one asymptomatic *Plasmodium* parasitaemia episode (red) or not (green) within 16 months of follow-up.

**Figure S12**. Gut fungal community structure. Relative abundance of phyla in children who experienced (yellow bars) or did not experience (blue bars) at least one malaria attack within 16 months of follow-up. (Figures inside the bars are the mean abundance of the phylum for each group).

**Figure S13**. Gut fungal community structure. Relative abundance of phyla in children who developed (blue bars) or did not develop (yellow bars) at least one asymptomatic *Plasmodium* parasitaemia episode within 16 months of follow-up. (Figures inside the bars are the mean abundance of the phylum for each group).
