# Supplementary material for: Gut microbiota influences Plasmodium falciparum malaria susceptibility
Source: New Microbes New Infect. 2025 Apr 14;65:101586. doi: 10.1016/j.nmni.2025.101586 (PMC12032372; doi:10.1016/j.nmni.2025.101586)
Supplement: Multimedia component 2 [file mmc2.docx]

| Characteristic | Age groups | | | | Total |
| --- | --- | --- | --- | --- | --- |
|  | 6 mo-4 y | 5-8 y | 9-11 y | 12-15 y |  |
| N (%) | 78 (26%) | 90 (30%) | 69 (23%) | 63(21%) | (n=300) |
| Malaria attack (n/%) |  |  |  |  |  |
| 0 | 58 (74%) | 58 (64%) | 36 (52%) | 41 (65%) | 193 (64%) |
| 1 | 13 (17%) | 12 (13%) | 23 (33%) | 14 (22%) | 62 (21%) |
| 2 | 7 (9%) | 15 (17%) | 6 (9%) | 3 (5%) | 31 (10%) |
| >2 | 0 | 5 (6%) | 4 (6%) | 5 (8%) | 14 (5%) |
| Asymptomatic parasitaemia episode (n/%) |  |  |  |  |  |
|  |  |  |  |  |  |
| 0 | 68 (87%) | 65 (72%) | 49 (71%) | 36 (57%) | 218 (73%) |
| 1 | 8 (10%) | 15 (17%) | 12 (18%) | 11 (18%) | 46 (15%) |
| 2 | 2 (3%) | 4 (4%) | 5 (7%) | 4 (6%) | 15 (5%) |
| >2 | 0 | 6 (7%) | 3 (4%) | 12 (19%) | 21 (7%) |

**Table S1**. Malaria outcomes by age group within 16 months of follow-up

**Table S2.** Logistic regression analysis of the association of age and gut bacterial and fungal community structure with the risk of at least one asymptomatic *Plasmodium* parasitaemia episode

| Variables | Univariate analysis | | Multivariate analysis | |
| --- | --- | --- | --- | --- |
|  | Odd Ratio (95%CI) | P value | Odd Ratio (95%CI) | P value |
| Age | 2.195 (1.383-3.485) | <10^-3^ | 2.102 (1.318-3.354) | 0.002 |
| **Bacteria community** | (82/300, 27.3%) |  |  |  |
| OTU Richness | 2.441 (1.163-5.123) | 0.018 | – | – |
| Chao-1 index | 1.427 (0.711-2.867) | 0.317 | – | – |
| Shannon index | 1.072 (0.556-2.068) | 0.835 | 5.398 (1.053 – 27.668) | 0.043 |
| Simpson index | 0.006 (0-105.518) | 0.308 | 0.00 (0 - 0.026) | 0.024 |
| **Fungi community** | (n=82/300, 27.3%) |  |  |  |
| OTU Richness | 1 (1 – 1) | 0.571 | – | – |
| Chao-1 index | 0.997 (0.988-1.006) | 0.524 | – | – |
| Shannon index | 1.187 (0.742 – 1.894) | 0.476 | – | – |
| Simpson index | 0.008 (0 – 2.558) | 0.102 | – | – |

Best-fitting transformation used: Age, ChaoLN, ShannonPuissance, Richness, SimpsonLN.

**Table S3.** Gut bacterial and fungal community structures according to the children’s age groups.

| Characteristics Mean (IC 95%) | Age groups | | | | Total |
| --- | --- | --- | --- | --- | --- |
|  | 6 mo-4 y | 5-8 y | 9-11 y | 12-15 y |  |
| N (%) | 78 (26%) | 90 (30%) | 69 (23%) | 63 (21%) | 300 (100%) |
| Bacteria Richness indices (n/%) | 77 (26%) | 90 (30%) | 67 (23%) | 62 (21%) | 296 (100%) |
| Observed OTU | 1025  (925.2-1125) | 1170 (1089-1252) | 1141  (1051-1232) | 1194  (1103-1284) |  |
| Chao-1 | 1321  (1193-1449) | 1496 (1375-1616) | 1320  (1220-1421) | 1323  (1204-1442) |  |
| Bacteria Diversity Indices | 77 (26%) | 90 (30%) | 67 (23%) | 62 (21%) | 296 (100%) |
| Shannon H | 4.79  (4.69-4.90) | 4.96  (4.88-5.03) | 4.86  (4.77-4.95) | 4.78  (4.69-4.84) |  |
| Simpson D | 0.93  (0.93-0.94) | 0.94  (0.93-0.94) | 0.93  (0.93-0.94) | 0.93  (0.92-0.93) |  |
| Fungi Richness Indices (n/%) | 76 (26%) | 90 (30%) | 68 (23%) | 62 (21%) | 296 (100%) |
| Observed OTU | 57.57  (53.12-62.01) | 53.52 (49.44-57.60) | 55.47  (50.94-60) | 57.50  (51.46-63.54) |  |
| Chao-1 | 76.77  (70.89-82.66) | 72.15 (66.85-77.45) | 76.03  (69.31-82.75) | 79.67  (71.61-87.73) |  |
| Fungi Diversity Indices | 76 (26%) | 90 (30%) | 68 (23%) | 62 (21%) | 296 (100%) |
| Shannon H | 1.71  (1.59-1.83) | 1.86  (1.75-1.98) | 1.97  (1.84-2.1) | 1.89  (1.75-2.03) |  |
| Simpson D | 0.65  (0.61-0.69) | 0.70  (0.66-0.73) | 0.72  (0.68-0.76) | 0.69  (0.65-0.73) |  |

**Table S4**. Gut bacterial and fungal community structure in children who experienced, or did not experience, at least one malaria attack within 16 months of follow-up.

| Mean (IC 95%) | Malaria attack (n=105) | No malaria attack (n=191) | P value |
| --- | --- | --- | --- |
| **Bacteria Richness indices** |  |  |  |
| Observed OTU | 1233 (1152 – 1313) | 1076 (1022 – 1129) | 0.001 |
| Chao-1 indice | 1457 (1352 – 1561) | 1330 (1257 – 1402) | 0.036 |
| **Bacteria Diversity Indices** |  |  |  |
| Shannon H | 4.895 (4.819 – 4.972) | 4.833 (4.777 – 4.889) | 0.141 |
| Simpson D | 0.9329 (0.9282 – 0.9377) | 0.9331 (0.9299 – 0.9363) | 0.800 |
| **Fungi Richness Indices** |  |  |  |
| Observed OTU | 55.57 (51.55 – 59.59) | 55.99 (53.13 – 58.84) | 0.566 |
| Chao-1 | 74.79 (69.49 – 80.09) | 76.38 (72.46 – 80.31) | 0.374 |
| **Fungi Diversity Indices** |  |  |  |
| Shannon H | 1.8 (1.682 – 1.918) | 1.838 (1.745 – 1.930) | 0.672 |
| Simpson D | 0.8571 (0.7891 – 0.9252) | 0.8429 (0.7909 – 0.8950) | 0.746 |

**Table S5.** Gut bacterial and fungal community structure in children who developed, or did not develop, at least one asymptomatic *Plasmodium* parasitaemia episode within 16 months of follow-up.

| Mean (IC 95%) | **Asymptomatic parasitaemia** (n=81) | **No parasitaemia episode** (n=215) | P value |
| --- | --- | --- | --- |
| **Bacteria Richness indices** |  |  |  |
| OTU Richness | 1217 (1130 – 1305) | 1099 (1046 – 1151) | 0.020 |
| Chao-1 | 1417 (1299 – 1536) | 1358 (1289 – 1428) | 0.490 |
| **Bacteria Diversity Indices** |  |  |  |
| Shannon H | 4.863 (4.773 – 4.952) | 4.852 (4.800 – 4.905) | 0.957 |
| Simpson D | 0.9308 (0.9251- 0.9365) | 0.9339 (0.9309 – 0.9369) | 0.395 |
| **Fungi Richness Indices** |  |  |  |
| OTU Richness | 55.52 (51.15 – 59.89) | 55.96 (53.21– 58.71) | 0.825 |
| Chao-1 | 74.19 (68.32 – 80.05) | 76.43 (72.69 – 80.17) | 0.814 |
| **Fungi Diversity Indices** |  |  |  |
| Shannon H | 1.852 (1.699 – 2.005) | 1.814 (1.731 – 1.896) | 0.740 |
| Simpson D | 0.8395 (0.7578 – 0.9212) | 0.8512 (0.8032 – 0.8991) | 0.856 |
